# Enhancing the precision of continuum robots in orthopedic surgery based on mechanical principles

**DOI:** 10.3389/fbioe.2024.1470069

**Published:** 2024-10-15

**Authors:** Tongtao Pang, Jinkui Liang, Zechen Lin, Xubin Zhang, Finxin Du

**Affiliations:** ^1^ Qilu Hospital Of Shandong University Dezhou Hospital, Dezhou, Shandong, China; ^2^ School of Mechanical Engineering, Shandong University, Jinan, China; ^3^ NMPA Key Laboratory for Quality Evaluation of Medical Materials and Biological Protective Devices, Shandong Institute of Medical Device and Pharmaceutical Packaging Inspection, Shandong, Jinan, China; ^4^ Key Laboratory of High-Efficiency and Clean Mechanical Manufacture of MOE, Shandong University, Jinan, China

**Keywords:** orthopedic surgery, notched continuum robot, mechanical modeling, beam deflection prediction model, beam constraint model, kinematics

## Abstract

**Introduction:**

In the field of orthopedic surgery, the notched continuum robot has garnered significant attention due to its passive compliance, making it particularly suitable for procedures in complex and delicate bone and joint regions. However, accurately modeling the notched continuum robot remains a significant challenge.

**Methods:**

This paper proposes a high-precision mechanical modeling method for the notched continuum robot to address this issue. The flexible beam deflection prediction model based on the beam constraint model is established. The force balance friction model considering internal friction is established. An accurate static model is obtained, which can accurately estimate the deformation and deflection behavior of the robot according to the input driving force. The kinematic model of the notched continuum robot based on the static model is established. This method achieves high accuracywhile ensuring computational efficiency.

**Results:**

Experimental results demonstrate that the static model's error is only 0.1629 mm, which corresponds to 0.25% of the total length of the continuum robot, which is 66 mm.

**Discussion:**

This research provides valuable insights into the modeling and control of continuum robots and holds significant implications for advancing precision in orthopedic surgery.

## 1 Introduction

 In recent years, minimally invasive surgery (MIS) is becoming increasingly important (Zhang et al., 2022). The application of surgical robots in the MIS field has developed rapidly, which has significantly improved the accuracy, stability and safety of surgery (Morris, 2005; Hofmair et al., 2024). Traditional rigid surgical robots, such as da Vinci (Crusco et al., 2014; Bhat et al., 2021), Revo-I (Lim et al., 2017), Flex (Schuler et al., 2015) and other surgical robot systems, have been widely used in minimally invasive surgeries. However, despite the success of traditional surgical robots in many surgical fields (Mehta et al., 2022), their rigid structures pose limitations during surgeries (Lin et al., 2024). Tibial plateau fractures are a common type of intra-articular fracture, accounting for approximately 1% of all fractures. The primary clinical features include joint surface collapse and displacement, often necessitating surgical intervention (Elsoe et al., 2015; Ramponi and McSwigan, 2018). However, the surgical space within the joint is complex and narrow, making it difficult for traditional rigid instruments to adapt flexibly, potentially leading to greater surgical trauma.

To overcome the limitations of rigid surgical instruments, researchers have developed a new type of surgical robot: the continuum robots (Zhang et al., 2022; Wei et al., 2023; Zhang et al., 2024a; Du et al., 2024). The continuum robot is a type of robot that is flexible and can imitate the movement of biological structures. Its design is inspired by the tentacles or tongues of animals in nature, such as elephant trunks and octopus tentacles (Robinson and Davies, 1999). Unlike traditional rigid robots, continuum robots can bend, twist and stretch during surgery, making them better suited to the complex anatomical structures inside the human body (Zhang et al., 2022). For example, in spinal endoscopy and arthroscopic minimally invasive surgery, the continuum robots can enter the target area through a tiny wound and accurately position and fix the implant, thereby reducing damage to surrounding tissues. In addition, the continuum robots can dynamically adjust its operation path under the guidance of real-time images, further improving the safety and effectiveness of the surgery (Ma et al., 2021).

Among continuum robots, the notched continuum robots have gradually become a research hotspot. Kutzer et al. (2011) designed a 6 mm tendon-driven notched arthroscopic tool. A bidirectional asymmetric rectangular notch design was adopted to achieve continuum bending by extruding the notch groove on the surface of the cylinder. The advantage of this design is that it simplifies the modeling of the continuum manipulator. The disadvantage is that the width and length of the notch are relatively small compared to the continuum itself, resulting in a smaller bending limit for the continuum. This means that more joints are required to achieve the same bending angle, necessitating greater driving force. York et al. (2015) designed a continuum manipulator with only a single-directional asymmetric rectangular notch for small-diameter wrist surgical tools, which is driven by tendons. The advantage of the design of a unidirectional asymmetric rectangular notch is that the moment manipulator from the tendon actuation point to the neutral bending plane of the flexible beam is longer, resulting in reduced tendon actuation forces for devices of comparable diameter. However, the significant drawback of this design is its inability to bend in both directions within a single plane. Although the overall rotation of the device can compensate for this limitation under current conditions, in the long term, the design of unidirectional asymmetric notched continuum robots will not meet the increasingly complex operational requirements of surgical robots. Eastwood et al. (2018) improved the design of unidirectional asymmetric notches by modifying the simple rectangular design to a trapezoidal design with rounded corners at the notch edges and introducing contact-assist devices. This aims to avoid stress concentration at the notch corners, increase joint stiffness, and alter the shape of the continuum robot during bending. However, this design increases the modeling difficulty of the robot and still only allows bending in one direction within a single plane.

In order to achieve precise operation of continuum robots in surgery, the modeling method is particularly important. The kinematic modeling of continuum robots can be divided into the following categories: segmented models (Olson et al., 2020), constant curvature models (Webster III and Jones, 2010), and numerical methods (Bieze et al., 2018). In segmented models, continuum robots are usually simulated as a combination of a series of flexible segments. These models usually use Euler-Bernoulli beam theory (Bauchau and Craig, 2009) or Timoshenko beam theory (Hutchinson, 2001) to describe the bending and twisting of each segment. These theories help researchers predict the shape and behavior of continuum robots. For example, Zhang et al. (2024b) established a static model using the Euler Bernoulli beam theory, greatly improving the accuracy of the model. Static analysis involves calculating the equilibrium state reached by the robot under the action of external and internal forces. This includes understanding the response of the robot under different loads, interface reactions, and preset postures. The influence of different factors, such as friction, gravity, and environmental constraints, on the static behavior of the continuum robot is studied. In the constant curvature model, the bending shape of the continuum robot is assumed to be an arc, that is, the bending curvature is constant. This simplifies the kinematic description of the robot and facilitates fast computation and real-time control. For example, Zhang et al. (Du et al., 2023) established an inverse kinematics model based on constant curvature and introduced Kepler elliptic curves, greatly improving computational efficiency.

In this paper, a continuum robot suitable for arthroscopic minimally invasive internal fixation of tibial plateau fractures is proposed. The flexible beam deflection prediction model based on beam constraint model and the force balance friction model are proposed. The static model and kinematic model of the notched continuum robot are established. The contributions of this paper are as follows:

• Based on the flexible beam deflection prediction model and force balance friction model of beam constraint model, the static model of the notched continuum robot is established. The error of the static model is only 0.1629 mm, accounting for 0.25% of the total length of the continuum robot.

• The kinematic model based on the statics is established. And an efficient inverse kinematics algorithm is designed using the fitting method.

• The changing laws of friction force and friction coefficient under different force angles during the bending process of the notched continuum robot are experimentally analyzed, so that the accurate friction coefficient is determined.

The rest of this paper is as follows. Section 2 describes structural design of the notched continuum robot. Section 3 establishes statics analysis of the notched continuum robot. Section 4 establishes static modeling of the notched continuum robot. Section 5 conducts experiments and results analysis. Section 6 summarizes the entire paper.

## 2 Structural design of the notched continuum robot

**FIGURE 1:**
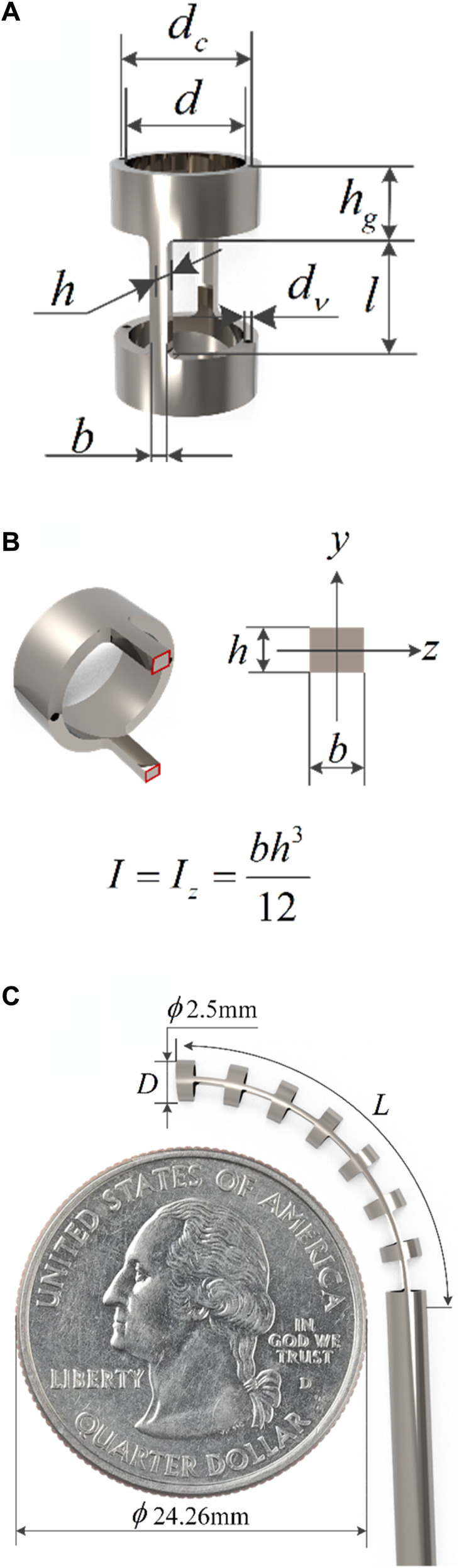
Structural diagram of the notched continuum robot. **(A)** Single joint. **(B)** Cross section of flexible beam. **(C)** Diagram of the continuum robot compared to a quarter.

When designing the robot’s structure, the aspect ratio, bending angle, inner and outer diameter dimensions, and biocompatibility of the flexible beam are considered to ensure that the robot could adapt to the surgical environment and accommodate surgical instruments. The configuration of the notched continuum robot is shown in Figure 1C. The robot is made of nitinol material. There are many rectangular notches of the same size on the nitinol tube, which are evenly and symmetrically distributed along the axis. There are two symmetrically distributed cable holes on the rigid disk, and their diameters are slightly larger than the diameter of the nitinol drive cables. Two nitinol drive cables are used to control the movement of the robot, and the robot can achieve bending movement in two directions in the same plane.

The structural parameters of the robot are shown in Table 1, and some of the parameters are described in Figures 1A, B. l , h and b are the length, the height and the width of the beam, respectively. $h_{g}$ is the height of the rigid disk. $d_{v}$ is the diameter of the cable hole. $d_{c}$ is the diameter of the circle where the cable hole is located. D , d and L are the outer diameter, the inner diameter and the length of the continuum robot, respectively.

**TABLE 1:** Structural parameter table of the notched continuum robot.

Parameters	Values	Parameters	Values	Parameters	Values
*l*	2 mm	$d_{v}$	0.15 mm	*L*	22 mm
*h*	0.25 mm	$d_{c}$	2.25 mm	Number of notches	15
*b*	0.3 mm	*D*	2.5 mm	Number of joints	7
$h_{g}$	1 mm	*d*	2 mm	Number of beams	7

## 3 Statics analysis

This chapter conducts a static analysis of the notched continuum robot based on the flexible beam deflection prediction model and the force balance friction model. When simplifying the structure, the following assumptions are based on: the friction between the drive cables and the continuum robot only occurs at the nodes; the influence of gravity on the robot is ignored; when the continuum robot bends to one side, the drive cable on the other side relaxes; the stress conditions at the ends of the two beams at each joint are exactly the same; the torsion of the beam is not considered; the deformation of the disk is ignored; the buckling of the beam caused by the axial load is ignored.

### 3.1 The flexible beam deflection prediction model

**FIGURE 2:**
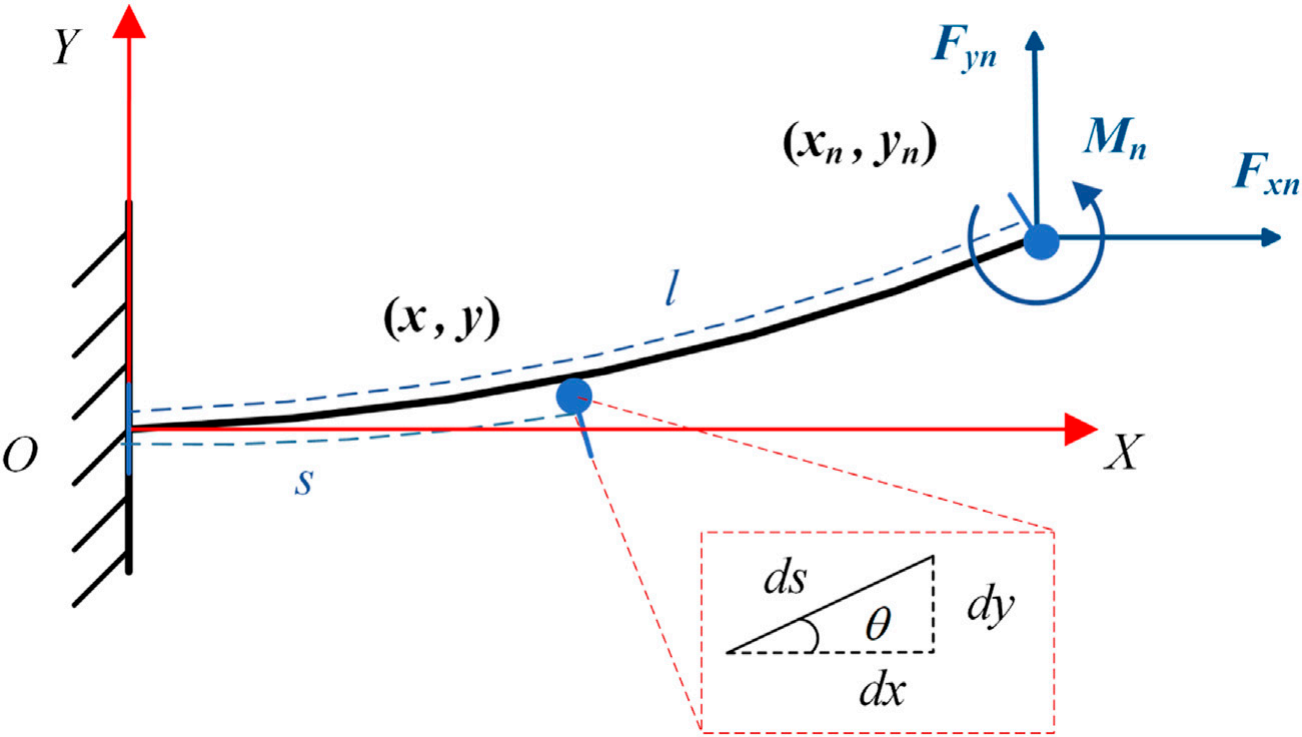
Flexible beam constraint model.

The flexible beam deflection prediction model based on beam constraint theory (Figure 2) has great advantages in accurately predicting the deflection behavior of the flexible beam in the intermediate deflection range. When the deflection is within 10% of the length of the flexible beam, the model can accurately capture the nonlinear deflection behavior related to the flexible beam. At the same time, the model is simple, closed-form and parameterized in equation form. In engineering applications, this advantage is very important for improving the calculation speed of the static model of the flexible beam and optimizing the real-time performance in the control system.

Transforming the classical Euler-Bernoulli beam equation into an ordinary differential equation yields:

$$\frac{d^{2}\theta }{ds^{2}}=-\frac{F_{y}}{EI}\left(\cos\theta \left(s\right)+\frac{F_{x}}{F_{y}}\sin\theta \left(s\right)\right)$$ (1)

In order to better describe the objective laws, the study of static models should be independent of the influence of physical model dimensions. Therefore, the dimensionless processing of Equation 1 yields Equation 2:

$$\frac{d^{2}\hat{\theta }}{d{\hat{s}}^{2}}=-f_{y}\cos\hat{\theta }\left(\hat{s}\right)-f_{x}\sin\hat{\theta }\left(\hat{s}\right)$$ (2)

Based on the beam constraint model, the beam end coordinates and deflection angle satisfy the relationship in Equations 3, 4:

$$\left[\begin{array}{c} \hat{y}\left(1\right)\\ \hat{\theta }\left(1\right) \end{array}\right]={\left(A-f_{x}B\right)}^{-1}\left[\begin{array}{c} f_{y}\\ m \end{array}\right]$$ (3)

$$\hat{x}\left(1\right)=-\frac{h^{2}}{12}f_{x}+{\left[\begin{array}{c} \hat{y}\left(1\right)\\ \hat{\theta }\left(1\right) \end{array}\right]}^{T}C\left[\begin{array}{c} \hat{y}\left(1\right)\\ \hat{\theta }\left(1\right) \end{array}\right]-f_{x}{\left[\begin{array}{c} \hat{y}\left(1\right)\\ \hat{\theta }\left(1\right) \end{array}\right]}^{T}D\left[\begin{array}{c} \hat{y}\left(1\right)\\ \hat{\theta }\left(1\right) \end{array}\right]+1$$ (4)

where parameters are shown in Equations 5, 6

$$f_{x}=\frac{F_{x}l^{2}}{EI};f_{y}=\frac{F_{y}l}{EI};m=\frac{Ml}{EI};\hat{s}\in \left[0,1\right]$$ (5)

$$A=\;\left[\begin{array}{cc} 12 & -6\\ -6 & 4 \end{array}\right];B=\left[\begin{array}{cc} 1.2 & -0.1\\ -0.1 & 2/15 \end{array}\right];C=\left[\begin{array}{cc} -0.6 & 1/20\\ 1/20 & -1/15 \end{array}\right];D=\left[\begin{array}{cc} 1/700 & -1/1400\\ -1/1400 & 11/6300 \end{array}\right]$$ (6)

Thus Equations 7, 8 can be obtained:

$$\hat{y}\left(1\right)=\frac{6lM\left(60EI-F_{x}l^{2}\right)+8F_{y}l^{2}\left(30EI-F_{x}l^{2}\right)}{3\sigma_{1}}$$ (7)

$$\hat{\theta }\left(1\right)=\frac{24lM\left(10EI-F_{x}l^{2}\right)+\;2F_{y}l^{2}\left(60EI-F_{x}l^{2}\right)}{\sigma_{1}}$$ (8)

where parameters are shown in Equations 9, 10

$$\sigma_{1}=240{\left(EI\right)}^{2}-104EIF_{x}l^{2}+3{F_{x}}^{2}l^{4}$$ (9)

$$\hat{x}\left(1\right)=g\left(l,M,E,I,F_{x},F_{y}\right)$$ (10)

### 3.2 The force balance friction model

**FIGURE 3:**
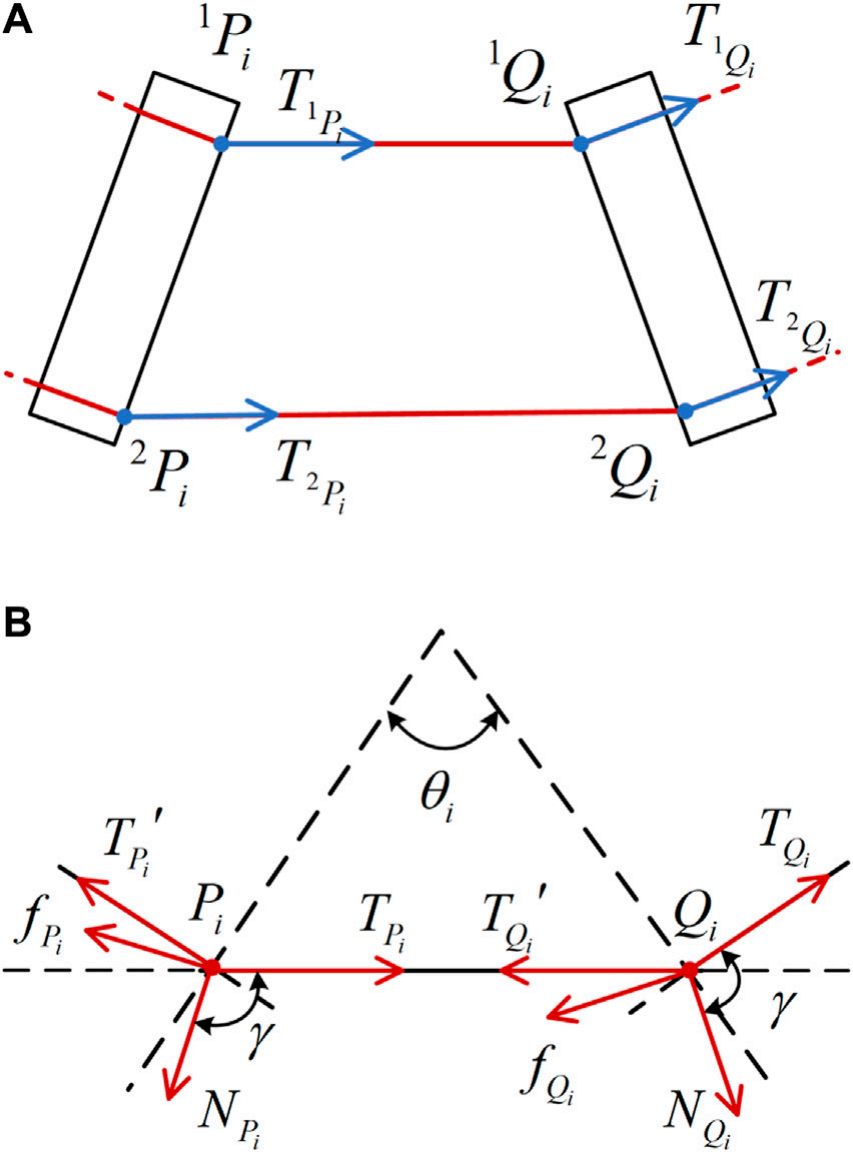
Force analysis. **(A)** Driving force at a single joint. **(B)** Forces on nodes.

The cable holes of each joint are denoted as nodes $Q_{i}$ and $P_{i}$ , and their force analysis is shown in Figure 3. Where N is the positive pressure generated by the driving cable on the nitinol tube, f is the friction force, and f=μN .

The Equation 11 is a mechanical analysis of node $Q_{i}$ . The following vector equilibrium equation is established at node $Q_{i}$ :

$$\vec{T_{Q_{i}}}=\vec{N_{Q_{i}}}+\vec{{T_{Q_{i}}}^{\prime }}+\vec{f_{Q_{i}}}$$ (11)

transforming it into the modulus equilibrium equation can be obtained as follows:

$$T_{Q_{i}}={T_{Q_{i}}}^{\prime }+f_{Q_{i}}$$ (12)

The static equilibrium equations are established in the $T_{Q_{i}}$ direction and its perpendicular direction respectively:

$$T_{1}\left(\gamma ,N_{Q_{i}},{T_{Q_{i}}}^{\prime }\right)=N_{Q_{i}}\left(\mu \sin\gamma -\cos\gamma \right)+{T_{Q_{i}}}^{\prime }\cos\left(\frac{\theta_{i}}{2}\right)-T_{Q_{i}}=0$$ (13)

$$T_{2}\left(\gamma ,N_{Q_{i}},{T_{Q_{i}}}^{\prime }\right)=N_{Q_{i}}\left(\sin\gamma +\mu \cos\gamma \right)-{T_{Q_{i}}}^{\prime }\sin\left(\frac{\theta_{i}}{2}\right)=0$$ (14)

among them, γ is the angle between the positive pressure $N_{Q_{i}}$ and the driving force, and $\theta_{i}$ is the bending angle $T_{Q_{i}}$ of the i -th joint of the continuum robot.

It can be obtained from Equation 12 that

$$T_{3}\left(\gamma ,N_{Q_{i}},{T_{Q_{i}}}^{\prime }\right)=\mu N_{Q_{i}}\cos\alpha +{T_{Q_{i}}}^{\prime }-T_{Q_{i}}=0$$ (15)

Introducing the tension loss coefficient k , which is shown in Equation 16:

$$k=\frac{{T_{Q_{i}}}^{\prime }}{T_{Q_{i}}}$$ (16)

From Equations 13–15, the following results can be obtained:

$$\left\{\begin{array}{c} k=\frac{2\mu^{2}{\sigma_{2}}^{2}+{\sigma_{2}}^{2}\pm 2\mu \sigma_{2}\sqrt{\mu^{2}\sigma_{2}+\sigma_{2}+1}+1}{{\sigma_{2}}^{2}+1}\;\\ \gamma =2\tan{\left(\frac{\sigma_{2}-\mu \mp \sqrt{\mu^{2}{\sigma_{2}}^{2}+{\sigma_{2}}^{2}+1}}{\mu \sigma_{2}-\mu +1}\right)}^{-1}\; \end{array}\right.$$ (17)

where parameter is shown in Equation 18

$$\sigma_{2}=\tan\left(\frac{\theta_{i}}{2}\right)$$ (18)

When the driving cable moves in the reverse direction, k<1 ; when the driving cable moves in the forward direction, k>1 . And the value of k is only related to $\theta_{i}$ . Similarly, the node $P_{i}$ also satisfies the above rule.

### 3.3 Static model

**FIGURE 4:**
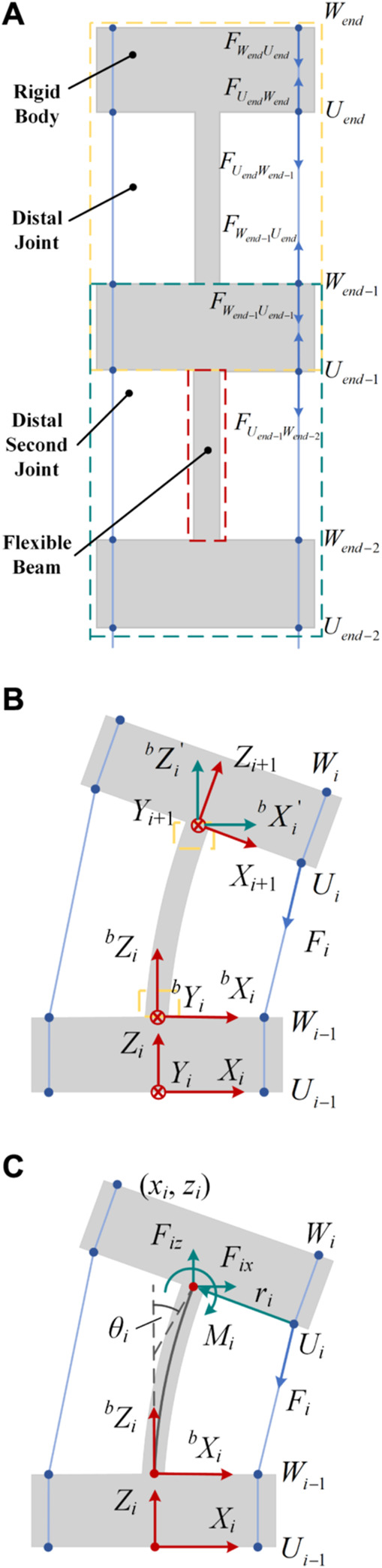
Static model analysis diagram of the notched continuum robot. **(A)** Force conditions of the robot’s distal joints. **(B)** Single joint statics coordinate system. **(C)** Equivalent load and deformation of a single-joint flexible beam.

The notched continuum robot can be considered as a series of flexible beams and rigid disks. As shown in Figure 4, each joint can be considered as consisting of two rigid disks and a flexible beam.

The force on the rigid disk at the distal joint satisfies the relationship as shown in Equation 19.

$$\vec{F_{W_{\mathrm{end}}U_{\mathrm{end}}}}=-\vec{F_{U_{\mathrm{end}}W_{\mathrm{end}}}}$$ (19)

Therefore, the external force on the distal joint is as shown in Equation 20.

$$\vec{F_{\mathrm{end}}}=\vec{F_{U_{\mathrm{end}}W_{\mathrm{end}}}}+\vec{F_{U_{\mathrm{end}}W_{\mathrm{end-1}}}}+\vec{F_{W_{\mathrm{end}}U_{\mathrm{end}}}}=\vec{F_{U_{\mathrm{end}}W_{\mathrm{end-1}}}}$$ (20)

The resultant external force on the distal second joint is as shown in Equation 21.

$$\vec{F_{\mathrm{end-1}}}=\vec{F_{W_{\mathrm{end-1}}U_{\mathrm{end}}}}+\vec{F_{W_{\mathrm{end-1}}U_{\mathrm{end-1}}}}+\vec{F_{U_{\mathrm{end-1}}W_{\mathrm{end-1}}}}+\vec{F_{U_{\mathrm{end-1}}W_{\mathrm{end-2}}}}+\vec{F_{\mathrm{end}}}=\vec{F_{U_{\mathrm{end-1}}W_{\mathrm{end-2}}}}$$ (21)

By generalization, the resultant force of the external force on any joint is as shown in Equation 22.

$$\vec{F_{i}}=\vec{F_{U_{i}W_{i-1}}}$$ (22)

The direction vector of $\vec{F_{i}}$ be obtained by coordinate matrix transformation as shown in Equation 23:

$$\vec{U_{i}W_{i-1}}=W_{i-1}-U_{i}={\left[\begin{array}{ccc} \frac{d}{2}\left(1-\cos\theta_{i}\right)-x_{i} & 0 & \frac{d}{2}\sin\theta_{i}-z_{i} \end{array}\right]}^{T}$$ (23)

Thus it can be obtained in Equation 24.

$$\vec{F_{i}}=F_{i}\frac{\vec{U_{i}W_{i-1}}}{\left\|\vec{U_{i}W_{i-1}}\right\|}$$ (24)

The equivalent forces applied to the free ends of the beam are:

$$F_{ix}=\frac{F_{i}\left(\frac{d}{2}\left(1-\cos\theta_{i}\right)-x_{i}\right)}{\sqrt{{\left(\frac{d}{2}\left(1-\cos\theta_{i}\right)-x_{i}\right)}^{2}+{\left(\frac{d}{2}\sin\theta_{i}-y_{i}\right)}^{2}}}$$ (25)

$$F_{iz}=\frac{F_{i}\left(\frac{d}{2}\sin\theta_{i}-z_{i}\right)}{\sqrt{{\left(\frac{d}{2}\left(1-\cos\theta_{i}\right)-x_{i}\right)}^{2}+{\left(\frac{d}{2}\sin\theta_{i}-z_{i}\right)}^{2}}}$$ (26)

The equivalent moment applied to the free end of the beam is

$$M_{i}=\vec{U_{i}{O_{i}}^{\prime }}\times \vec{F_{i}}=\frac{F_{i}d\left(\left(\frac{d}{2}\left(1-\cos\theta_{i}\right)-x_{i}\right)\sin\theta_{i}+\left(\frac{d}{2}\sin\theta_{i}-z_{i}\right)\cos\theta_{i}\right)}{2\sqrt{{\left(\frac{d}{2}\left(1-\cos\theta_{i}\right)-x_{i}\right)}^{2}+{\left(\frac{d}{2}\sin\theta_{i}-z_{i}\right)}^{2}}}$$ (27)

where $\vec{U_{i}{O_{i}}^{\prime }}$ is the moment arm of $\vec{F_{i}}$ , which is shown in Equation 28.

$$\vec{U_{i}{O_{i}}^{\prime }}={\left[\begin{array}{ccc} \frac{d}{2}\cos\theta_{i} & 0 & -\frac{d}{2}\sin\theta_{i} \end{array}\right]}^{T}$$ (28)

Based on the force balance friction model, the transmission relationship of the driving force $F_{i}$ between the joints can be obtained as shown in Equation 29.

$$\left\{\begin{array}{c} F_{1}=k_{1}F_{0}i=1\;\\ F_{i}=k_{i}k_{i-1}F_{i-1}i>1\; \end{array}\right.$$ (29)

Based on the beam constraint model, the deflection angle and coordinates of the beam end can be obtained:

$$\theta_{i}=\frac{24lM_{i}\left(10EI-F_{ix}l^{2}\right)+\;2F_{iz}l^{2}\left(60EI-F_{ix}l^{2}\right)}{240-104EIF_{ix}l^{2}+3{F_{ix}}^{2}l^{4}}l$$ (30)

$$z_{i}=\frac{6lM_{i}\left(60EI-F_{ix}l^{2}\right)+8F_{iz}l^{2}\left(30EI-F_{ix}l^{2}\right)}{3\left(240{\left(EI\right)}^{2}-104EIF_{ix}l^{2}+3{F_{ix}}^{2}l^{4}\right)}l$$ (31)

$$x_{i}=g\left(l,M_{i},E,I,F_{ix},F_{iz}\right)\cdot l$$ (32)

In order to solve the deflection angle and the end coordinates, this paper uses the fixed-point iteration method for calculation. Based on MATLAB, programming is performed to solve and build a modeling algorithm for the notched continuum robot, which is shown in Algorithm 1.

Algorithm 1 Static model algorithm of notched continuum robot.

**Input:** Initial ${deflection}^{0}\theta ={\left[\begin{array}{cccc} 0 & 0 & \cdots & 0 \end{array}\right]}^{T}$ , initial position ${coordinates}^{0}x={\left[\begin{array}{cccc} l & l & \cdots & l \end{array}\right]}^{T}{,}^{0}z={\left[\begin{array}{cccc} 0 & 0 & \cdots & 0 \end{array}\right]}^{T}$ , initial driving ${force}^{0}F$ , initial Iterations i=0 , initial error ε(0)=1 , accuracy threshold $\zeta =10^{-5}$ ; **Output:** The end position x , y and deflection angle θ of each joint beam
1: **While** ε(i)>ζ

2: ${Substitute}^{i}\theta$ into Equation 17 to solve the force loss ${coefficient}^{i}k$

3: ${Substitute}^{i}k$ ${and}^{0}F$ into Equation 29 to solve the driving ${force}^{i}F$ applied to each joint
4: ${Solve}^{i}F_{x}{,}^{i}F_{y}$ ${and}^{i}M$ in the local coordinate system according to Equations 25–27
5: ${Solve}^{i+1}\theta {,}^{i+1}z$ , ${and}^{i+1}x$ using Equations 30–32
6: ε(i+1)=θi+1−θi

7: i=i+1

8: **End While**
9: Get the deformation ${amount}^{i+1}\theta {,}^{i+1}z$ , ${and}^{i+1}x$ of the beam under the action of the ${force}^{0}F$

## 4 Kinematic modeling

In this section, the piecewise constant curvature (PCC) model of the notched continuum robot is established. Then, based on the static model, the chain rule is used to solve the homogeneous coordinate matrix of the end position of the continuum robot, thereby obtaining the position and posture of the robot in the workspace. The inverse kinematics model is constructed by the data fitting method, and the relationship between the length of the drive cables and the end coordinates of the continuum robot is established.

### 4.1 Forward kinematic modeling based on PCC

The PCC assumption is a classic hypothesis in the kinematic modeling of continuum robots. It uses the idea of differentials to divide the continuum robot into small segments. Within each small segment, the continuum robot is approximated an arc with the same curvature. This assumption simplifies the kinematic model of the continuum robot and effectively reduces the complexity of modeling the continuum robot.

**FIGURE 5:**
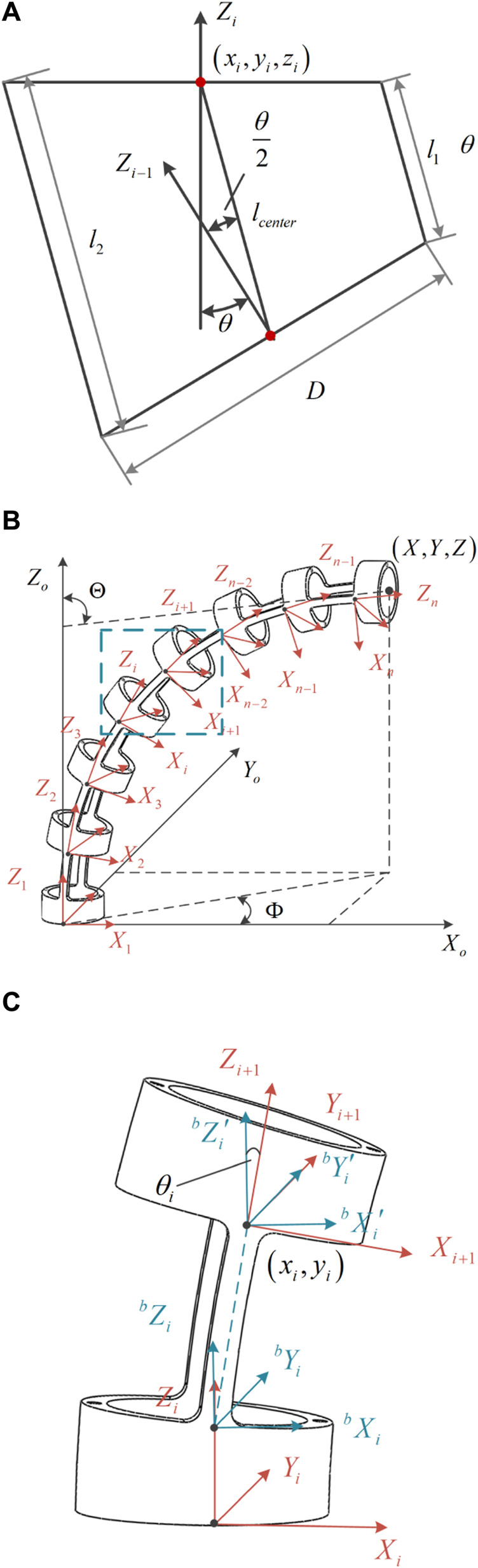
The deformation diagram and coordinate system establishment for the notched continuum robot are illustrated as follows. **(A)** Deformation diagram of a single joint of the notched continuum robot. **(B)** Kinematic coordinate system of the continuum manipulator. **(C)** Coordinate system of a single continuum joint beam and joint coordinates.

Based on the PCC assumption, the deformation of a single joint of the notched continuum robot can be simplified as shown in Figure 5A. According to the analysis of Figure 5A, the deflection angle θ of each joint of the continuum robot can be obtained as shown in Equation 33.

$$\theta =2\arcsin\left(\frac{l_{2}-l_{1}}{2D}\right)$$ (33)

According to the PCC assumption, the relationship is shown in Equations 34, 35.

Θ=nθ (34)

$$L_{k}=nl_{k}+C,\left(k=1,2\right)$$ (35)

where C is the height constant of the rigid disk.

According to Figure 5B, the transformation matrix between joint i and joint i+1 is given by Equation 36.

$$T_{i}^{i+1}=Trans\left(x_{i},y_{i},z_{i}\right)Rot\left(Y,\theta \right)Trans\left(0,0,C\right)$$ (36)

Define the end of the continuum robot as $P={\left[x,y,z\right]}^{T}$ , then $T=\left[\begin{array}{cc} R_{3\times 3} & P\\ 0 & 1 \end{array}\right]$ . Given the length of the driving cable, the end position of the notched continuum robot can be solved by Equation 37.

$$\left[\begin{array}{c} x\\ y\\ z \end{array}\right]=\left[\begin{array}{c} f_{x}\left(L_{1},L_{2},D,C\right)\\ f_{y}\left(L_{1},L_{2},D,C\right)\\ f_{z}\left(L_{1},L_{2},D,C\right) \end{array}\right]$$ (37)

### 4.2 Forward kinematic modeling based on static model

In the context of minimally invasive surgery, the influence of factors such as end load and friction cannot be ignored, and the PCC assumption is difficult to achieve. Therefore, this paper establishes the kinematic model of the notched continuum robot based on the static model.

The coordinate system for the rectangular notched continuum robot is established as shown in Figure 5B. In the world coor.

Dinate system, the total deflection angle of the continuum robot’s bending deformation is Θ , the twist angle around the axis- $Z_{o}$ is Φ , and the end coordinates of the robot are denoted as (X,Y,Z) .

Assuming that the homogeneous transformation matrix between two adjacent joints is $T_{i}^{i+1}$ , the forward kinematics of the continuum robot is solved using a modular approach. The coordinate transformation process of the continuum robot with a rectangular cutout in the middle of a single joint is shown in Figure 5C. The homogeneous transformation matrix between two adjacent joints is shown in Equation 38.

$$\begin{array}{c} T_{i}^{i+1}=Trans\left(0,0,h_{g}\right)Trans\left(x_{i},y_{i},z_{i}\right)Rot\left(Y,\theta_{i}\right)\\ =\left[\begin{array}{cccc} \cos\left(\theta_{i}\right) & 0 & \sin\left(\theta_{i}\right) & x_{i}\\ 0 & 1 & 0 & y_{i}\\ -\sin\left(\theta_{i}\right) & 0 & \cos\left(\theta_{i}\right) & z_{i}+h_{g}\\ 0 & 0 & 0 & 1 \end{array}\right] \end{array}$$ (38)

According to the chain rule, the homogeneous coordinate matrix of the end position of the continuum robot can be obtained, thereby obtaining the position and posture of the end of the continuum manipulator in the workspace, which is shown in Equation 39.

$$T=T_{1}^{2}T_{2}^{3}\ldots T_{i}^{i+1}\ldots T_{n-2}^{n-1}T_{n-1}^{n}Rot\left(Y,\Phi \right)$$ (39)

**FIGURE 6:**
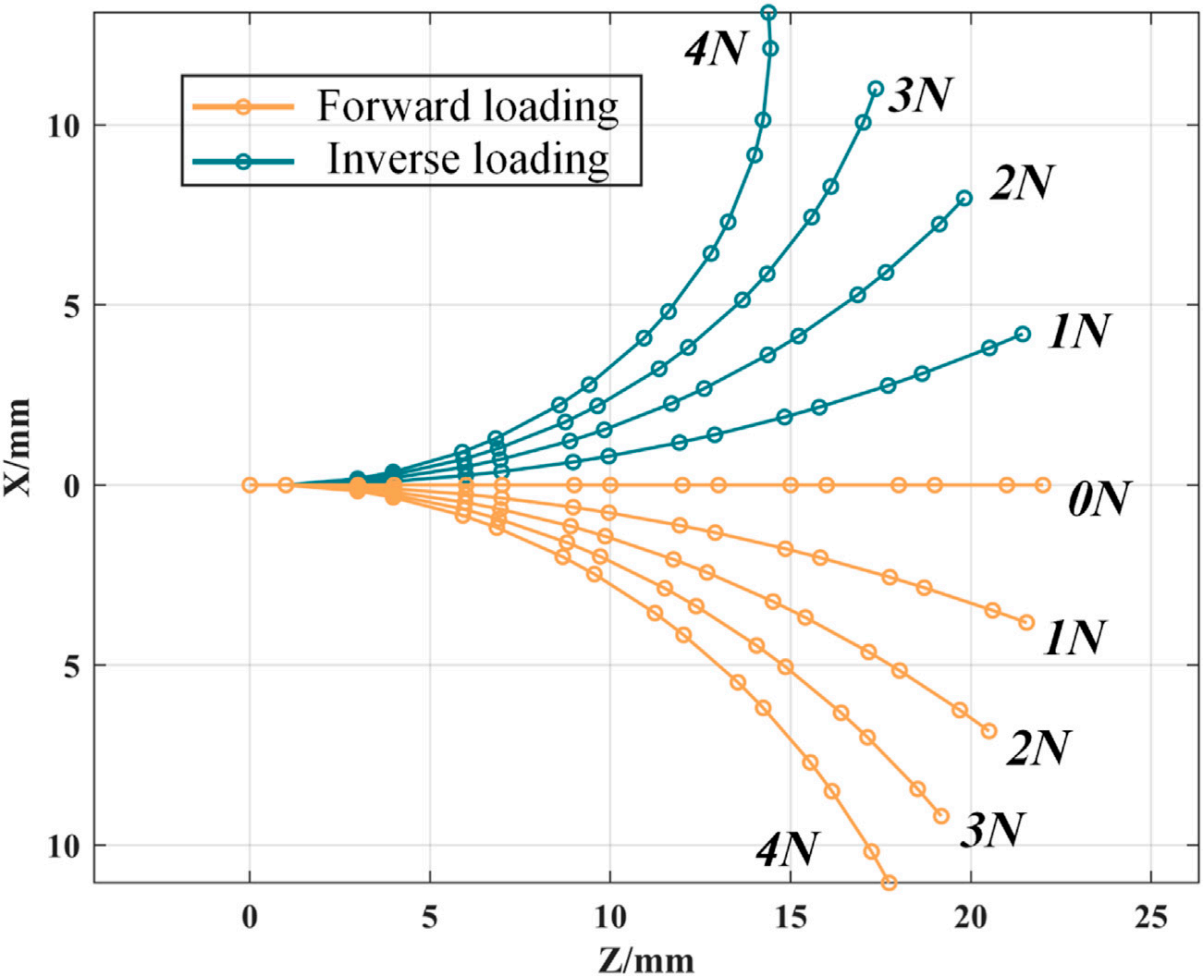
The notched continuum robot deflects in a single plane.

According to the static model, the force loss coefficients of the continuum robot are different when it is loaded forward and reversely, so the deformation curves in space are also different. As shown in Figure 6, the bending deformation of the continuum robot in a single plane is simulated in MATLAB.

### 4.3 Inverse kinematic modeling

The variable in the driving space of the cable-driven continuum robot proposed in this paper is the length of the driving cable. The cable length of the continuum robot can be calculated by the coordinates between the nodes in the static model in Equation 40.

$$L=\overset{n}{\underset{i=1}{\sum }}\left\|\vec{U_{i}W_{i-1}}\right\|+n\cdot h_{g}$$ (40)

When bending within a single plane, the length of the driving cable of the rectangular notched continuum robot is fitted with the end coordinates X and Z .

$$\left\{\begin{array}{c} L=a_{1}x^{6}+b_{1}x^{5}+c_{1}x^{4}+d_{1}x^{3}+e_{1}x^{2}+f_{1}x+g_{1}\;\\ L=a_{2}z^{6}+b_{2}z^{5}+c_{2}z^{4}+d_{2}z^{3}+e_{2}z^{2}+f_{2}z+g_{2}\; \end{array}\right.$$ (41)

**FIGURE 7:**
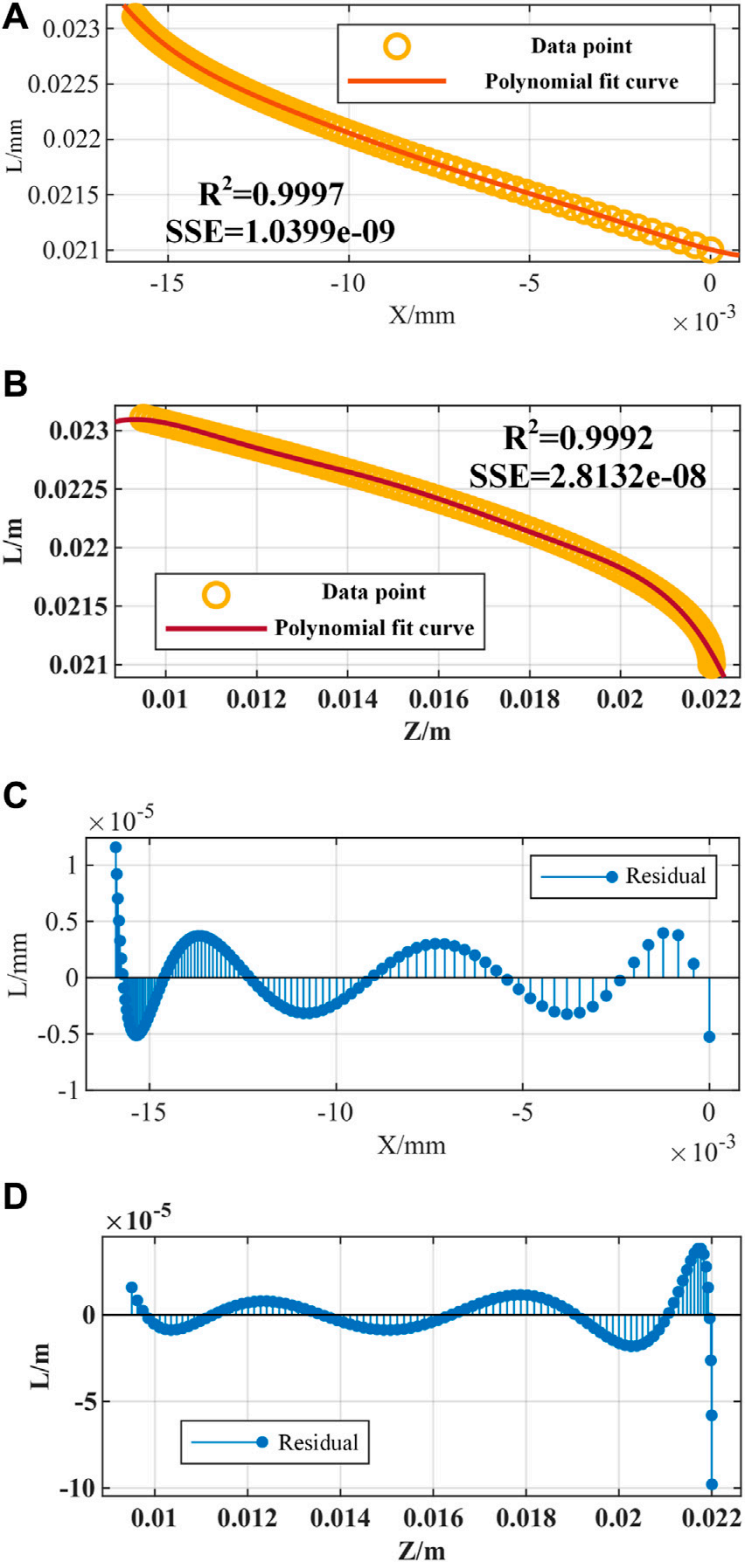
Inverse kinematics fitting simulation of the notched continuum robot. **(A)** Fitting curve of the driving cable length *L* and the end *X* coordinate. **(B)** The fitting curve of the driving cable length *L* and the end *Z* coordinate. **(C)** The fitting residual of *L* and *X*. **(D)** The fitting residual of *L* and *Z*.

As shown in Figure 7, the maximum residual error of the fitting curve does not exceed 0.1 mm, which is within the allowable error range.

According to the forward kinematics model, the end coordinates of the continuum robot are only related to the length of the driving cable. When the continuum robot undergoes an overall rotation as shown in Equation 42:

$$\Phi =\arctan\left(\frac{y}{x}\right)$$ (42)

$$R=\sqrt{x^{2}+y^{2}}$$ (43)

Replacing x in Equation 41 with R in Equation 43, the inverse kinematics model of the notched continuum robot in three-dimensional space is obtained.

## 5 Experiment and result analysis

In this section, the force balance friction model and the static model are experimentally validated. By measuring the friction force and friction coefficient of the continuum robot at different angles between the driving cable and the applied force, the relationship between these parameters and the bending angle of the continuum robot is obtained. The effectiveness of the static model is validated by comparing the bending curves of the notched continuum robot under different driving forces with the theoretical model.

### 5.1 Friction coefficient determination

**FIGURE 8:**
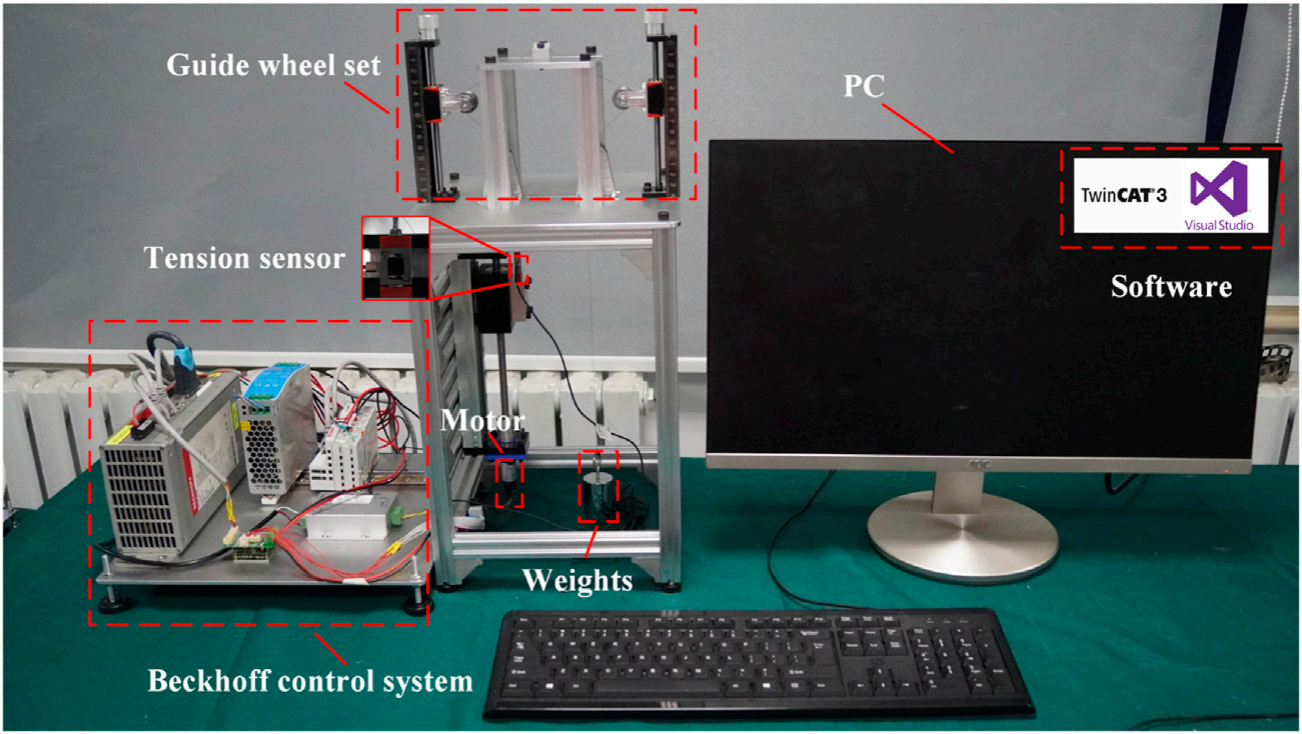
Experimental platform for measuring friction force and friction coefficient of single-segment notched continuum.

The experimental platform is shown in Figure 8, which is used to measure the friction force and friction coefficient of a single-segment notched continuum. The drive cable of the continuum robot is made of nickel-titanium alloy cable. The drive cable passes through the single-segment notched continuum and is connected to the weight and tension sensor through the guide wheels on both sides. The control program is implemented in TwinCAT3, and all sensor data are collected in Visual Studio (ver 3.1.0). The tension sensor needs to be calibrated and the system friction needs to be balanced before the experiment.

**FIGURE 9:**
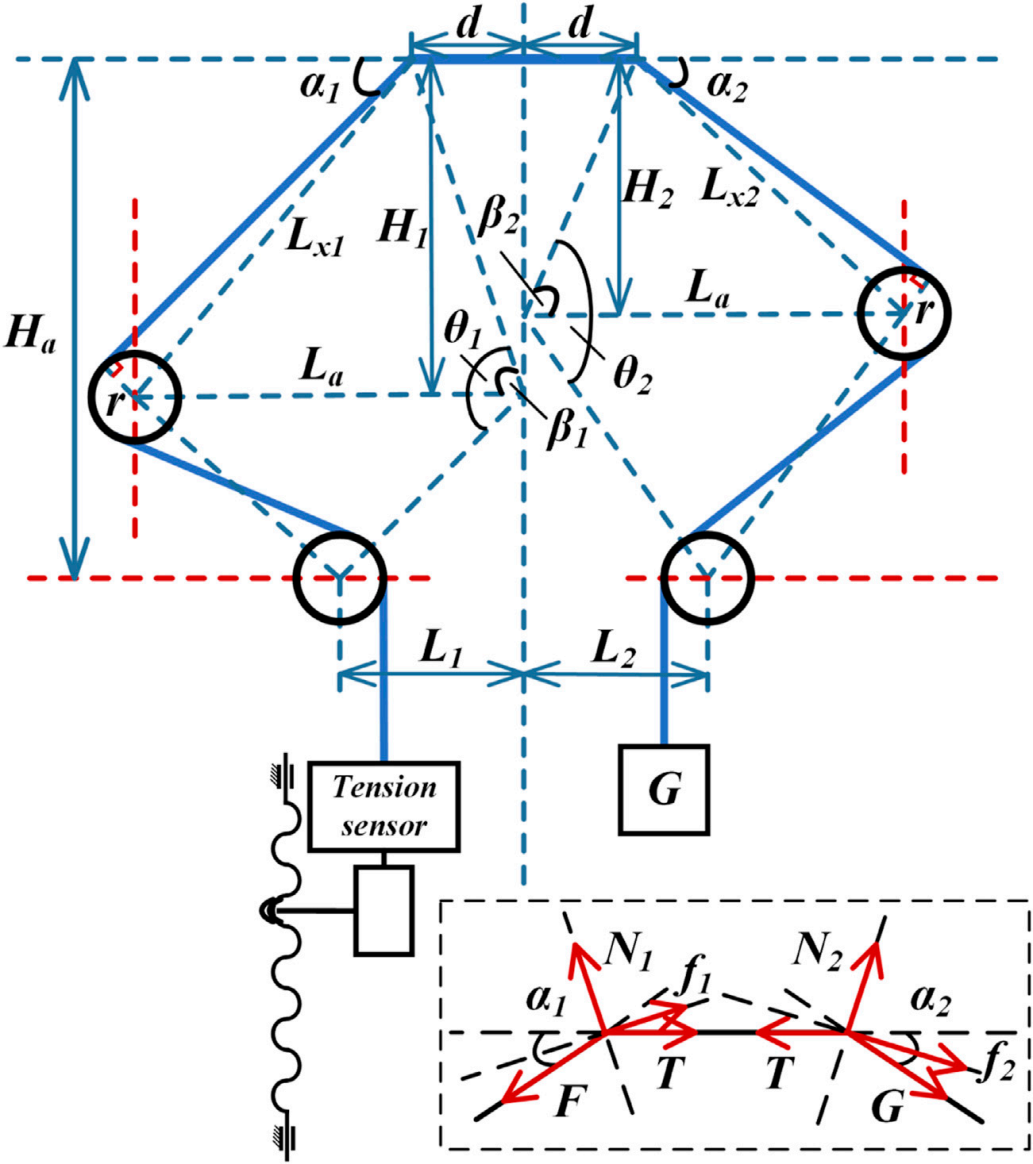
Geometric parameter solution diagram for the friction coefficient measurement experiment.

To ensure the accuracy of the angle measurements, the geometric parameters during the experiment are derived from the experimental schematic shown in Figure 9. According to Equation 44, the angles corresponding to different heights are calculated, and the friction forces at 13 different angles are measured.

$$\left\{\begin{array}{c} \alpha_{i}=\pi -\arctan\frac{H_{i}}{d}-\arcsin\frac{L_{a}\sin\beta_{i}}{L_{xi}}-\arcsin\frac{r}{L_{xi}}\;\\ \theta_{i}=\pi -\arctan\frac{d}{H_{i}}-\arctan\frac{L_{i}}{H_{a}-H_{i}}\;\\ \beta_{i}=\theta_{i}-\frac{\pi }{2}+\arctan\frac{L_{i}}{H_{a}-H_{i}}\;\\ L_{xi}=\sqrt{L_{a}^{2}+H_{i}^{2}+d^{2}-2L_{a}\sqrt{H^{2}+d^{2}}\cos\beta_{i}}\; \end{array}\right.$$ (44)

Figure 9 shows the forces acting on both ends of the notched continuum during the experiment. The analysis yields the following:

$$\left\{\begin{array}{c} F\cos\left(\frac{\alpha_{1}}{2}\right)=\mu N_{1}+T\cos\left(\frac{\alpha_{1}}{2}\right)\;\\ F\sin\left(\frac{\alpha_{1}}{2}\right)+T\sin\left(\frac{\alpha_{1}}{2}\right)=N_{1}\; \end{array}\right.$$ (45)

$$\left\{\begin{array}{c} T\cos\left(\frac{\alpha_{2}}{2}\right)=\mu N_{2}+mg\cos\left(\frac{\alpha_{2}}{2}\right)\;\\ mg\sin\left(\frac{\alpha_{2}}{2}\right)+T\sin\left(\frac{\alpha_{2}}{2}\right)=N_{2}\; \end{array}\right.$$ (46)

The combined Equations 45, 46 and data verification can be used to solve the experimentally obtained the friction coefficient, which is shown in Equation 47.

$$\mu_{2}=\;\frac{\left(F+mg\right)\sin\left(\frac{\alpha_{1}+\alpha_{2}}{2}\right)-\sqrt{{\left(mg+F\right)}^{2}\sin^{2}\left(\frac{\alpha_{1}+\alpha_{2}}{2}\right)-{\left(F-mg\right)}^{2}\sin\left(\alpha_{1}\right)\sin\left(a_{2}\right)}}{2\left(F-mg\right)\sin\left(\frac{\alpha_{1}}{2}\right)\sin\left(\frac{\alpha_{2}}{2}\right)}$$ (47)

**FIGURE 10:**
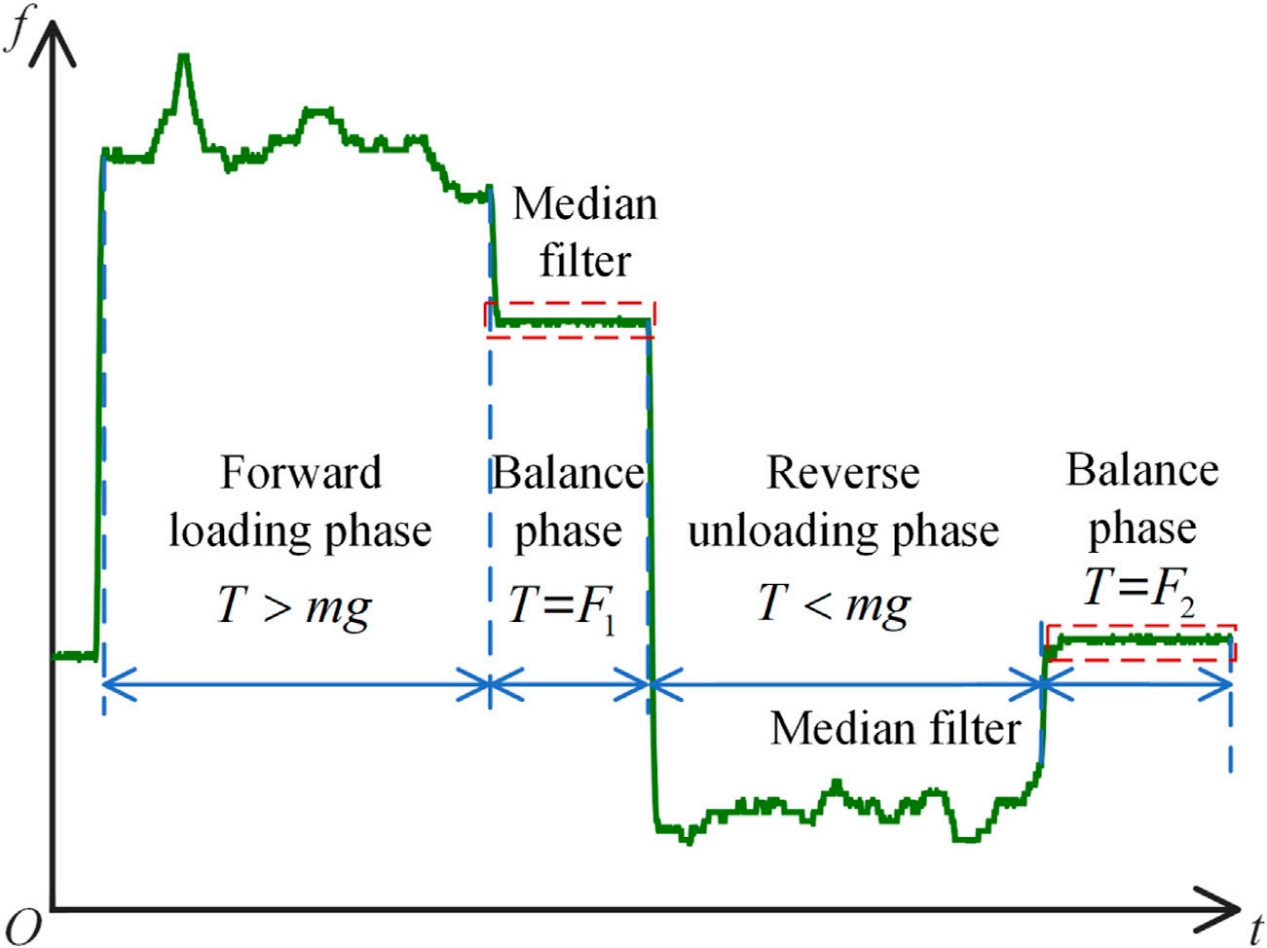
Four stages of friction change during the experiment.

As shown in Figure 10, during the experiment, the friction force undergoes the stages of forward loading phase, balance phase, reverse unloading phase, and balance phase. The average of 20 data sets is taken as the friction force for the single-segment notched continuum at each angle. The experimentally measured friction force f is shown in Equation 48.

$$f=\frac{F_{1}-F_{2}}{2}$$ (48)

**FIGURE 11:**
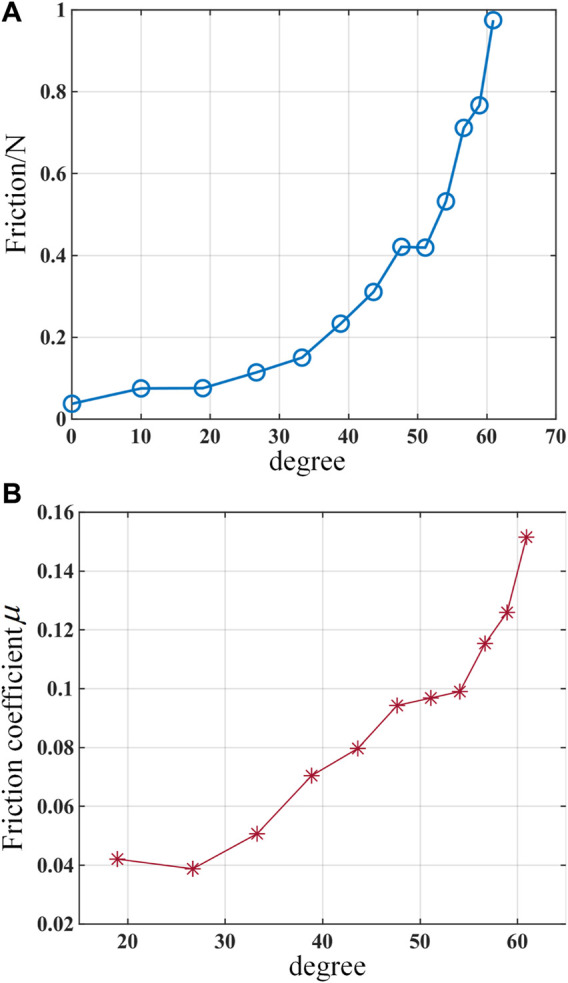
Experimental results of a single-segment notched continuum. **(A)** Curve of friction force changing with the angle between the driving cable and the continuum robot. **(B)** Curve of friction coefficient changing with the angle between the driving cable and the continuum robot.

According to the experimental results shown in Figure 11, the friction force between the driving cable and the cable hole of the notched continuum robot tends to increase with the increase of the angle between the driving cable and the continuum robot, and the friction coefficient increases with the increase of the angle within a small range.

### 5.2 Static model validation

**FIGURE 12:**
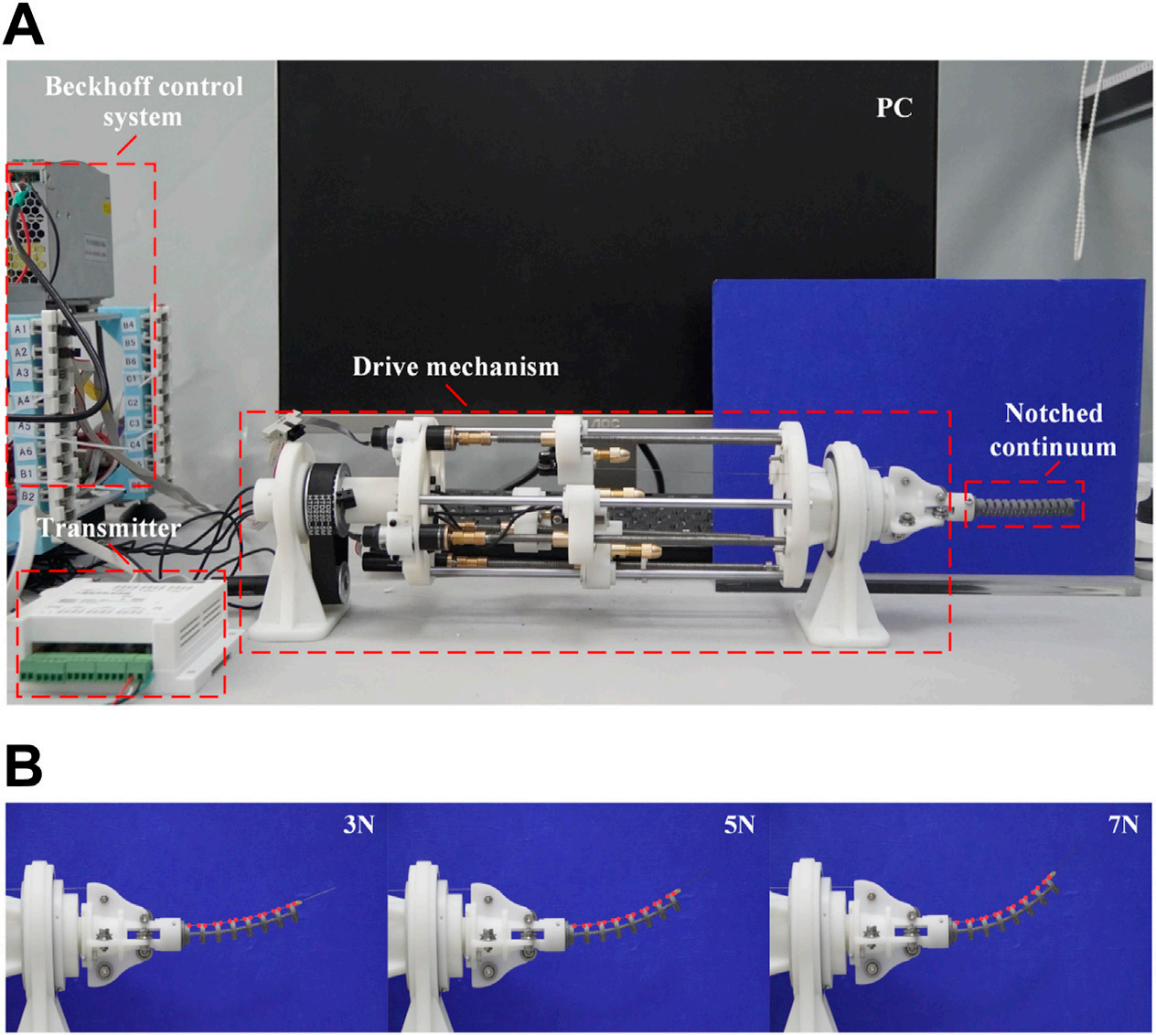
The experiment for the static model verification. **(A)**Experimental platform. **(B)** Deformation of the notched continuum robot under different driving forces.

The experimental platform used to verify the static model established in this study is shown in Figure 12A. Machine vision software Vision Master (ver 3.1.0) is used to process images and extract deformation characteristic points of the continuum robot within the bending plane. Post-experiment data processing is completed using MATLAB. The notched continuum nylon samples were fabricated using 3D printing. The key structural parameters and material properties of the samples are listed in Table 2.

**TABLE 2:** Geometric parameters and physical properties of the notched continuum robot.

Parameters	Values	Parameters	Values
External diameter	11 mm	Cutting height	6 mm
Inner diameter	7 mm	Total length	66 mm
Beam length	6 mm	Beam number	7
Beam width	1.5 mm	Materials	Nylon
Beam depth	2 mm	Bending modulus	1800 MPa

**FIGURE 13:**
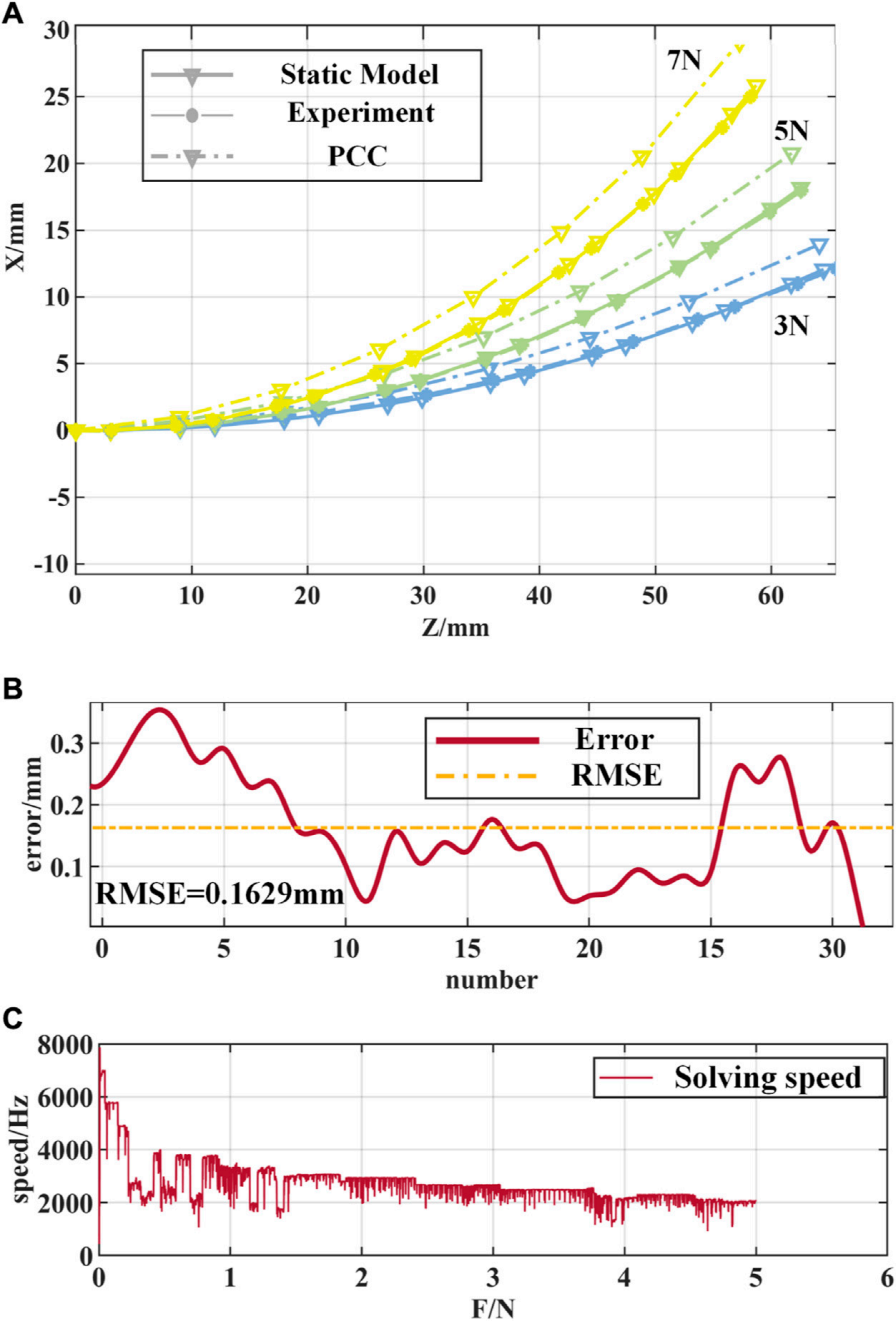
Comparison of experimental results of the notched continuum robot. **(A)** Comparison of the curved shapes of the static model, the PCC model and the experiment. **(B)** Error curves of the static model and experimental feature points. **(C)** Solution speed of the static model of the notched continuum robot.

As shown in Figure 12B, the deformation of the notched continuum robot under the driving force of 3N, 5N, and 7N is analyzed. The red dots marked in the figure are the deformation feature points identified by Vision Master. The coordinates of the feature points in the bending plane are processed based on MATLAB, and the bending curve of the continuum is plotted and compared with the theoretical results as shown in Figure 13A.

From the experimental results in Figure 13A, it can be seen that the static model proposed in this paper is closer to the experimental results in terms of the curved shape and end position of the notched continuum robot than the PCC assumption. As shown in Figure 13B, the root mean square error (RMSE) of the feature point position of the notched continuum robot based on the static model is 0.1629 mm, accounting for 0.247% of the total length of the continuum, and the error is significantly lower than the error result under the PCC assumption. As shown in Figure 13C, the solution speed of the static model of the notched continuum robot decreases with the increase of the driving force, but it is maintained above 1,000 Hz, which verifies the high efficiency of the static model constructed in this paper, which is of great significance for the real-time control of the notched continuum robot.

## 6 Conclusion

This paper proposes to apply the notched continuum robot to arthroscopic minimally invasive internal fixation of tibial plateau fractures. The flexible beam deflection prediction model and the force balance friction model based on beam constraint model are proposed. The static model and kinematic model of the notched continuum robot are established respectively. Compared with the traditional PCC assumption, the modeling method proposed in this paper has a faster solution speed and higher accuracy. Specifically, the modeling method proposed in this paper effectively analyzes the distribution of friction force and the loss of driving force in the continuum, and establishes a force balance friction model that changes with the deformation of the continuum. Based on the beam constraint model, combined with the deflection prediction model and the force balance friction model, this paper constructs an efficient static model solution algorithm. The kinematic model based on the static model is established, and the bending behavior of the robot in a single plane is plotted. Through the fitting method, this paper also simplifies the inverse kinematics calculation to ensure that the maximum fitting residual does not exceed 0.01 mm in the normal working space. In order to further verify the method in this paper, the friction change law under different force angles is analyzed by the friction coefficient measuring instrument, which proves that the friction force and friction coefficient increase with the increase of the bending angle. Finally, the static model is verified by experiments to have higher accuracy in predicting the bending shape and end position of the continuum robot, with the root mean square error of the feature point position being only 0.1629 mm, and the single solution speed being above 1,000 Hz. The results of this study have a high reference value for the modeling and control of the continuum robot and are of great significance to the development of minimally invasive orthopedic surgery.

## Data Availability

The original contributions presented in the study are included in the article/supplementary material, further inquiries can be directed to the corresponding authors.
